# Neurotology symptoms at referral to vestibular evaluation

**DOI:** 10.1186/1916-0216-42-55

**Published:** 2013-11-26

**Authors:** Kathrine Jáuregui-Renaud, Aralia Gutierrez-Marquez, Leticia Viveros-Rentería, Verónica Ramos-Toledo, Fátima Gómez-Alvarez

**Affiliations:** 1 Unidad de Investigación Médica en Otoneurología, Planta baja del Edificio C-Salud en el Trabajo Centro Médico Nacional siglo XXI, IMSS, Av. Cuauhtémoc 330, Colonia Doctores, CP 06720, México, DF, México; 2 Departamento de Audiología y Otoneurología, HG CMN La Raza, Instituto Mexicano del Seguro Social, México DF, México

**Keywords:** Vestibular, Vertigo, Dizziness

## Abstract

**Background:**

Dizziness-vertigo is common in adults, but clinical providers may rarely diagnose vestibular impairment and referral could be delayed. To assess neurotology symptoms (including triggers) reported by patients with peripheral vestibular disease, during the year just before their referral to vestibular evaluation.

**Methods:**

282 patients with peripheral vestibular disease and 282 control subjects accepted to participate. They had no middle ear, retinal, neurological, psychiatric, autoimmune or autonomic disorders. They reported their symptoms by a standardized questionnaire along with their anxiety/depression symptoms.

**Results:**

Patients were referred after months or years from the onset of their symptoms, 24% of them reported frequent falls with a long clinical evolution; 10% of them reported no vertigo but instability related to specific triggers; 86% patients and 12% control subjects reported instability when moving the head rapidly and 79% patients and 6% control subjects reported instability when changing posture. Seven out of the 9 symptoms explored by the questionnaire allowed the correct classification of circa 95% of the participants (Discriminant function analysis, p < 0.001). High blood pressure, dyslipidemia and anxiety/depression symptoms showed a mild correlation with the total score of symptoms (multiple R^2^ =0.18, p < 0.001).

**Conclusions:**

Late referral to vestibular evaluation may underlie a history of frequent falls; some patients may not report vertigo, but instability related to specific triggers, which could be useful to prompt vestibular evaluation. High blood pressure, dyslipidemia and anxiety/depression symptoms may have a mild influence on the report of symptoms of vestibular disease in both, patients and control subjects.

## 

Vertigo-dizziness is a common symptom in adults [1], and it is among the most common reasons that a patient presents to emergency departments [2,3]. It may be due to vestibular or psychiatric causes in more than 70% of cases [4]. However, clinical providers may rarely diagnose vestibular impairment and may not be aware of the appropriate treatment [5]. Then, referral could be delayed [6], even after several medical consultations [7]. The medical history and the profile of symptoms may be helpful to identify patients who require vestibular evaluation [8]. However, there is little research on the specific symptoms reported by patients with peripheral vestibular disease, just at referral to vestibular evaluation [6,9,10]. Since in patients with balance disorders, psychological or psychiatric symptoms may interfere with self-report of symptoms, simultaneous assessment of symptoms of anxiety/depression is recommended [11,12].

The aim of this study was to assess neurotology symptoms (including triggers) reported by patients with peripheral vestibular disease, during the year just before their referral to vestibular evaluation, compared to those perceived by control subjects with no medical history of vestibular disease, adjusted by their general characteristics and the report of symptoms of anxiety/depression.

After approval by the Research and Ethics Committee, within an institutional healthcare system, 282 patients (17 to 80 years old, 150 females/132 males) and 282 control subjects (19 to 80 years old, 163 females/119 males) were selected consecutively. They had similar education and employment status; none of them had evidence or medical record of middle ear, retinal, neurological, autoimmune or autonomic disorders, or submission to psychiatric care or psychopharmacological treatment.

After a clinical evaluation, the following questionnaires were administered for self-report:

A standardized Neurotology questionnaire including the 9 symptoms described on Table 1, with a KR20 (Kuder-Richardson formula 20) of 0.75 and an intra-class rank correlation coefficient of 0.9 [13]; the total symptom score was calculated as the sum of the individual scores of each item, in a scale from 0 to 10, as follows:

– Items: 1, 2, 3, 4, 5 and 8, No = 0 and Yes = 1

– Item 6, No = 0 and Yes = 1 only when the frequency reported was ≥1 per week;

– Item 7, No = 0 and Yes = 1 only when the frequency reported was ≥1 per month;

– Item 9, No = 0 and Yes = 2.

**Table 1:** Frequency of symptoms by general diagnosis of 282 patients with peripheral vestibular disease and 282 control subjects

**Variables by general diagnosis**	**Vestibular lesion**		**Cochleo-vestibular disease, other than Meniere’s disease**		**Meniere’s disease**	**Benign paroxismal positional vertigo**	**Total vestibular patients**	**Controls**
	**Unilateral**	**Bilateral**	**Unilateral**	**Bilateral**				
*Number of patients*	*(n = 93)*	*(n = 29)*	*(n = 42)*	*(n = 82)*	*(n = 13)*	*(n = 23)*	*(n = 282)*	*(n = 282)*
*Characteristic (Median, Quartile 1 - Quartile 3)*								
Years of age	37 (37–53)	40 (34–48)	40.5 (33–54)	58 (49–69)	37 (36–47)	50 (43–58)	46 (35–57)	45 (37–53)
Months of clinical evolution	2 (0.2-12)	24 (6–72)	18 (2–36)	24 (4–60)	4 (1–12)	4 (2–12)	8 (1–24)	-
Total score	7 (5–8)	7 (5–8)	7 (5–9)	7 (5–9)	9 (6–10)	7 (6–8)	7 (5–8)	0 (0–1)
*Neurotology symptom (Number, percentage)*								
1. Instability when walking on uneven surfaces	58 (62%)	18 (62%)	26 (62%)	56 (68%)	8 (61%)	12 (52%)	178 (63%)	20 (7%)
2. Instability when walking in the dark	59 (63%)	22 (76%)	29 (69%)	55 (67%)	8 (61%)	13 (56%)	186 (66%)	27 (9%)
3. Instability when moving the head rapidly	83 (89%)	27 (93%)	34 (81%)	63 (77%)	12 (92%)	23 (100%)	242 (86%)	35 (12%)
4. Instability when changing posture rapidly	75 (80%)	23 (79%)	30 (71%)	61 (74%)	11 (84%)	23 (100%)	223 (79%)	29 (10%)
5. Instability when looking at moving objects	61 (65%)	16 (51%)	23 (55%)	54 (66%)	11 (84%)	14 (60%)	179 (63%)	17 (6%)
6. Frequent stumbles ( ≥ 1/week)	31 (33%)	14 (48%)	21 (50%)	44 (54%)	9 (69%)	8 (35%)	127 (45%)	18 (6%)
7. Frequent falls (≥ 1/month)	10 (10.7%)	8 (28%)	9 (21%)	32 (39%)	5 (38%)	3 (13%)	67 (24%)	4 (1%)
8. Dizziness	87 (93.5%)	28 (96%)	36 (86%)	76 (92%)	13 (100%)	20 (87%)	260 (93%)	47 (16%)
9. Spinning sensation (vertigo)	87 (93.5%)	24 (83%)	34 (81%)	70 (85%)	13 (100%)	23 (100%)	251 (89%)	12 (4%)

The 12-items General Health Questionnaire (GHQ-12) was scored in a scale from 0 to 12, using the “GHQ method” of 0-0-1-1, where a total score ≥3 was considered as evidence of anxiety/depression symptoms [14].

The 20-items questionnaire of depression was scored in a scale from 20 to 80, which was calculated as the sum of the individual scores of each item (from 1 to 4), where a total score ≥36 was considered as evidence of symptoms of anxiety/depression [15].

According to data distribution, statistical analysis was performed with a significance level of 0.05 (Statistica, Statsoft Inc., Tulsa), using “t” test, Mann Whitney “U” test, *X*^2^, Kruskall Wallis ANOVA, Discriminant function analysis and ANCoVA.

## Patients

The time elapsed since the onset of the symptoms was from 2 days to 28 years. Twenty six percent of the patients had systemic high blood pressure, 11% diabetes mellitus, 8% dyslipidemia and 5% had degenerative arthritis. 182 patients (66%) reported anxiety/ depression symptoms. The general diagnoses after vestibular evaluation (with image studies when required) are shown on Table 1. Patients with unilateral vestibular disease were younger and had a clinical evolution shorter than patients in most of the other subgroups, while patients with bilateral vestibulo-cochlear disease (apart from Meniere’s disease) were older and had a clinical evolution longer than patients from most of the other subgroups (Kruskall Wallis ANOVA, p < 0.001).

Apart from dizziness (93%) and vertigo (89%), the most frequent symptoms were (Table 1): instability when moving the head rapidly (86%) and when changing posture (79%); which were reported by 100% of the patients with Benign Paroxysmal Positional Vertigo. In all patients, instability in these situations was reported more frequently than instability triggered by any other situation (*X*^2^, p < 0.01). Frequent falls were reported by 67 patients (24%), with a mean age of 53 years (S.D. 15 years), and a median evolution time of 2 years (Quartile 1 = 4 months & Quartile 3 = 5 years), which was longer than the evolution time reported by patients with no falls (“t” test, p < 0.001). The median of the total score of the questionnaire of all patients was 7, from 2 to 10 (Q1 = 5 & Q3 = 9) (Table 1). Covariance analysis on the total score showed a significant contribution of the age (beta = 0.15, 95% C.I. 0.017-0.28) and the report of anxiety/depression symptoms (beta = 0.21, 95% C.I. 0.07-0.35) (multiple R^2^ = 0.09, p < 0.001), with no influence of any other characteristic.

## Control subjects

Five percent of them had systemic high blood pressure, 4% diabetes mellitus, 1% dyslipidemia and 1% had degenerative arthritis. Fifty nine subjects (21%) reported anxiety/depression symptoms. The most frequent symptoms reported were dizziness (16%) and instability when moving the head rapidly (12%); 13 (4%) subjects reported vertigo and 3 (1%) reported frequent falls (Table 1). The subjects who reported vertigo were aged 19 to 64 years, and the subjects who reported falls were aged 22 to 51 years. The median of the total score of the questionnaire of all control subjects was 0 (Q1 = 0 & Q3 = 1). Covariance analysis showed that the employment status (beta = 0.21, 95% C.I. 0.04-0.38) and the report of anxiety/ depression symptoms (beta = 0.44, 95% C.I. 0.17-0.77) had an influence on the total score (multiple R^2^ = 0.13, p < 0.001), with no influence of any other characteristic.

## Comparisons between patients and control subjects

The frequency of each neurotology symptom and the total score were significantly different between the two groups, as well as the frequency of anxiety/ depression symptoms (“t” test for proportions and Mann Whitney “U” test, p < 0.01). Discriminant function analysis showed that the combination of seven out of the 9 neurotology symptoms allowed the correct classification of 93.9% of the patients and 96.4% of the control subjects (squared Malahanobis distance = 19.5, p <0.001), the symptoms with no significant contribution were stumbles and falls. The total score allowed the correct classification of 94.3% of the patients and 99.3% of the control subjects (squared Malahanobis distance = 13.8, p < 0.001). The 16 patients with vestibular disease who were misclassified were aged 23 to 76 years, none of them had Meniere’s disease or Benign Paroxysmal Positional Vertigo; they had an evolution time from 2 days to 25 years and 9 of them (56%) did not report vertigo, but dizziness or instability. The 2 control subjects who were misclassified were aged 38 and 53 years and had a total score of 4.

Multivariate analysis to identify the influence of the general characteristics of all participants (n = 564) on the total score, was significant for systemic high blood pressure (beta = 0.20, 95% C.I. 0.12-0.28), dyslipidemia (beta = 0.12, 95% C.I. 0.05-0.20) and the report of anxiety/depression symptoms (beta = 0.28, 95% C.I. 0.20-0.36) (multiple R^2^ = 0.18, p < 0.001).

Although evidence has shown that short-term duration of symptoms at referral (< 6 months) combined with programs of exercises may influence the prognosis positively [16], as well as that a proportion of unexplained fallers attending an Emergency Department may suffer from vestibular impairment [17,18], the results of this study are consistent with previous reports showing that patients with peripheral vestibular disease may be referred to specialized evaluation when they already suffer from chronic imbalance and may have fallen. Additionally, the importance of high blood pressure, dyslipidemia and symptoms of anxiety/depression when assessing dizziness-vertigo, may be emphasized.

In this study, the report of instability during movement is consistent with the vestibular function to transduce orientation and reorientation of the head in space. Consistently, these two triggers were reported by all the patients with Benign Paroxysmal Positional Vertigo. The report of these symptoms by some control subjects, even suggest that they may have not been able to recall a previous vestibular dysfunction. Additionally, a lower frequency to report instability while walking “on uneven surfaces” or “in the dark” or “while looking at moving objects”, suggests that, in such situations, sensory integration may have allowed better stability.

In order to interpret or extrapolate the findings of this study, several limitations should be considered: the cross sectional design, the recording of symptoms just at the time of referral, the limited information available of symptoms of anxiety/depression; and the inclusion of control subjects who may have not been able to recall a subtle vestibular dysfunction.

In conclusion, chronic imbalance related to delayed referral of patients with peripheral vestibular disease may underlie the occurrence of frequent falls. Patient with peripheral vestibular disease may not report vertigo but instability related to specific triggers, which could be early investigated to prompt vestibular evaluation.

## Competing interests

The authors declare that they have no competing interests.

## Authors’ contributions

KJR have made substantial contributions to conception and design of the study, acquisition of data, analysis and interpretation of data, drafting the manuscript and revising it critically. AGM, LVR and VRT have made substantial contributions to acquisition of data, interpretation of data and revising the manuscript critically. FGA have made substantial contributions to acquisition of data and revising the manuscript critically. All authors read and approved the final manuscript.
